# Role of transcription factors and chromatin modifiers in driving lineage reprogramming in treatment-induced neuroendocrine prostate cancer

**DOI:** 10.3389/fcell.2023.1075707

**Published:** 2023-01-12

**Authors:** Amritha Sreekumar, Sharanjot Saini

**Affiliations:** Department of Biochemistry and Molecular Biology, Augusta University, Augusta, GA, United States

**Keywords:** transcription factors, epigenetics, neuroendocrine prostate cancer, chromatin, lineage plasticity

## Abstract

Therapy-induced neuroendocrine prostate cancer (NEPC) is a highly lethal variant of prostate cancer that is increasing in incidence with the increased use of next-generation of androgen receptor (AR) pathway inhibitors. It arises *via* a reversible trans-differentiation process, referred to as neuroendocrine differentiation (NED), wherein prostate cancer cells show decreased expression of AR and increased expression of neuroendocrine (NE) lineage markers including enolase 2 (ENO2), chromogranin A (CHGA) and synaptophysin (SYP). NEPC is associated with poor survival rates as these tumors are aggressive and often metastasize to soft tissues such as liver, lung and central nervous system despite low serum PSA levels relative to disease burden. It has been recognized that therapy-induced NED involves a series of genetic and epigenetic alterations that act in a highly concerted manner in orchestrating lineage switching. In the recent years, we have seen a spurt in research in this area that has implicated a host of transcription factors and epigenetic modifiers that play a role in driving this lineage switching. In this article, we review the role of important transcription factors and chromatin modifiers that are instrumental in lineage reprogramming of prostate adenocarcinomas to NEPC under the selective pressure of various AR-targeted therapies. With an increased understanding of the temporal and spatial interplay of transcription factors and chromatin modifiers and their associated gene expression programs in NEPC, better therapeutic strategies are being tested for targeting NEPC effectively.

## 1 Introduction

Prostate cancer (PCa) is a leading cause of cancer incidence amongst men in US, with an estimated 268,490 new cases in 2022. It is one of the leading causes of cancer-related deaths among males, with an estimated 34,500 deaths in 2022 (Siegel et al., 2022). Prostate cancer is a hormone-dependent cancer that relies on androgens acting *via* binding to its receptor, androgen receptor (AR) (Knudsen and Scher, 2009). Considering the crucial role of AR signaling, inhibition of AR signaling *via* ablating androgens is the goal of first line of PCa treatment, referred to as androgen deprivation therapy (ADT). ADT results in cancer regression initially. However, in a significant fraction of patients, 2–3 years after ADT, the disease progresses to castration-resistant prostate cancer (CRPC) (Shen and Abate-Shen, 2010). The treatment options for CRPC are limited though over last several years, several new agents have been introduced in the clinics for treating non-metastatic and metastatic CRPC such as second generation of AR pathway inhibitors (API) enzalutamide (MDV3100/ENZ) and abiraterone (ABI) (Knudsen and Scher, 2009; Scher et al., 2012). Though these agents have contributed to better survival rates, drug resistance is a major clinical challenge. Drug resistance results from heterogeneous molecular mechanisms such as AR bypass signaling or complete AR independence (Watson et al., 2015; Culig, 2017). A subset of API-resistant tumors undergo a reversible lineage trans-differentiation known as neuroendocrine differentiation (NED), that results in altered expression of lineage markers. As a result of NED, PCa cells show decreased expression of AR and increased expression of neuroendocrine (NE) lineage markers including enolase 2 (ENO2), chromogranin A (CHGA) and synaptophysin (SYP) (Aggarwal et al., 2014; Aggarwal and Small, 2014). These PCa variants, referred to as therapy-induced neuroendocrine prostate cancer (NEPC), are highly aggressive, often metastasizing to soft tissues such as liver, lung and central nervous system despite low serum PSA levels relative to disease burden (Aggarwal et al., 2014). Therefore, NEPC is associated with poor survival rates (Aggarwal et al., 2014). Therapy-induced NEPC may encompass a spectrum of histological states ranging from adenocarcinomas with mixed NE histology to pure small cell neuroendocrine carcinoma (SCNC), which is similar to small cell cancers of other organs such as small cell lung cancer (SCLC) (Epstein et al., 2014). Normal human prostate can be reprogrammed to SCNC using a common set of defined oncogenic drivers, namely dominant negative p53 (TP53DN), myrAkt1, RB1-shRNA, c-Myc and Bcl-2 (Park et al., 2018). NEPC, like SCLC and other small cell neuroendocrine cancers (SCNC) exhibit common morphological and histological features such as high nuclear to cytoplasm ratios, granular chromatin and frequent mitotic figures (Klimstra et al., 2015). NEPC along with other SCNC tumors share genome-wide expression, methylation and copy number expression patterns and have common vulnerabilities that were reported to be similar to that of hematological malignancies (Balanis et al., 2019).

## 2 Clonal evolution of neuroendocrine prostate cancer: Genetics and epigenetics

**FIGURE 1:**
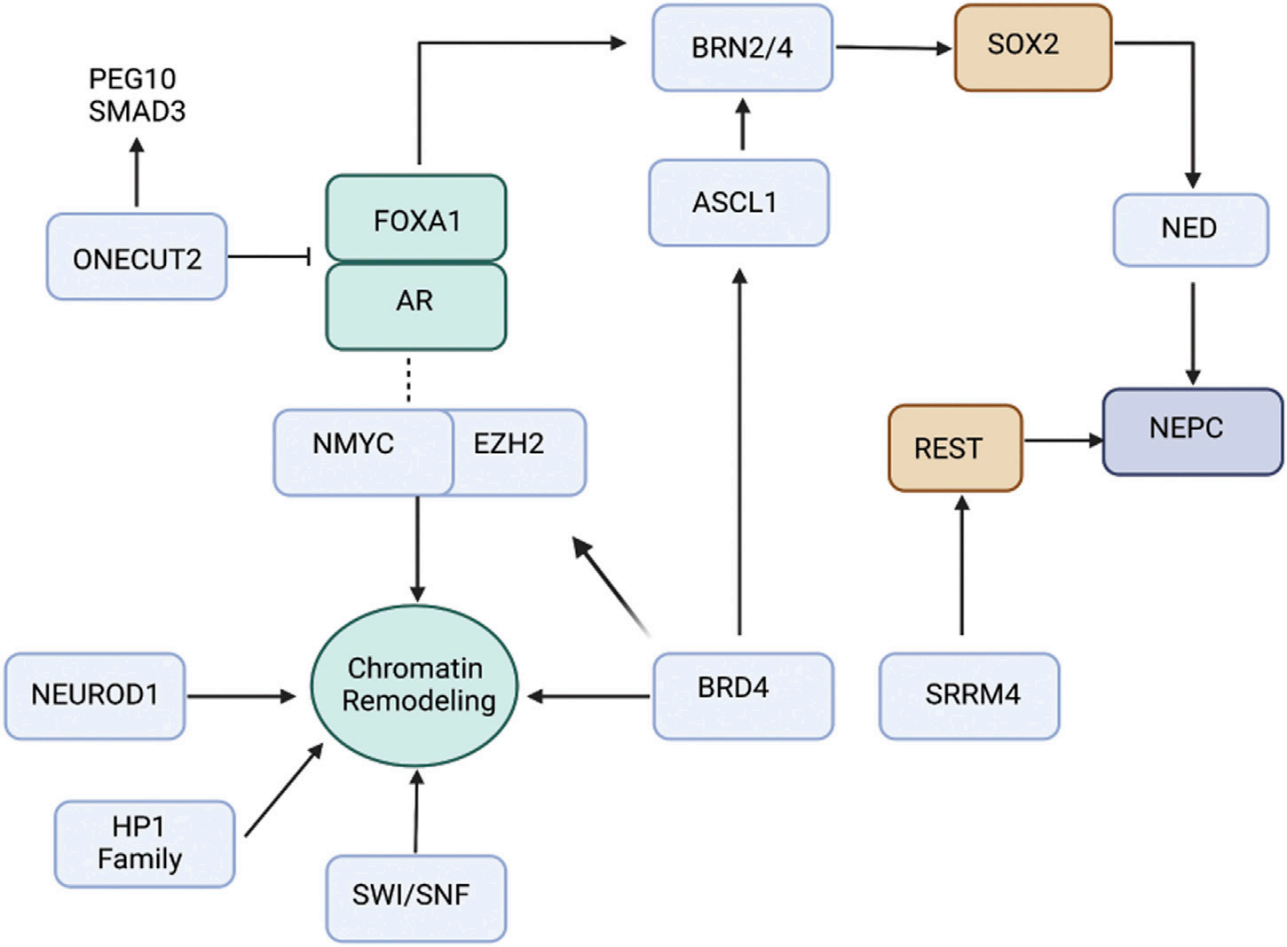
Schematic view of the interplay between transcription factors and chromatin regulators driving NEPC. ONECUT2 is a direct repressor of AR signaling, thereby promoting NEPC. ASCL1 is pioneering transcription factor controlling expression of neuronal genes. EZH2 is the histone methyltransferase that is pivotal in driving NEPC. Recent data suggest that association of EZH2 with AR drive reprogramming of AR cistrome that leads to expression of neuronal-associated gene programs. POU-domain TFs BRN2 and BRN4 play critical role in regulating SOX2 driving neuroendocrine differentiation. Several other factors including NEUROD1, BRD4, SWI/SNF and HP1 family drive epigenetic reprograming/chromatin remodeling leading to NEPC.

It has been realized that therapy-induced NEPC represents a continuum of treatment-induced changes at molecular level resulting from a series of genetic and epigenetic alterations (Beltran et al., 2011; Lotan et al., 2011; Beltran et al., 2016; Dardenne et al., 2016; Maina et al., 2016). Analyses show that PCa NE states are derived *via* clonal evolution from CRPC-adenocarcinomas (Beltran et al., 2016). The key genetic events driving this transition include loss of the tumor suppressors retinoblastoma (*RB1*) and mutation or loss of tumor protein 53 (*TP53*) (Tan et al., 2014; Beltran et al., 2016). Mouse models support that dual loss of *TP53* and *RB1* are important steps in the development of poorly differentiated NE tumors of the prostate (Zhou et al., 2006). Normal human prostate epithelial cells could be transformed into SCNC *via* expression of *RB1* shRNA and dominant negative *p*53 (Park et al., 2018). In addition, phosphatase and tensin homolog (*PTEN*) loss (Beltran et al., 2011; Lotan et al., 2011; Beltran et al., 2016; Dardenne et al., 2016; Maina et al., 2016) and frequent *TMPRSS2-ERG* gene rearrangements (Lotan et al., 2011) have been reported in NEPC. Furthermore, EZH2 overexpression and amplifications of *NMYC* and Aurora Kinase A (AURKA) (Beltran et al., 2011; Beltran et al., 2016; Dardenne et al., 2016; Maina et al., 2016) are cardinal alterations that have been associated with NEPC. AURKA is a cell cycle kinase that stabilizes N-Myc oncoprotein and prevents N-Myc degradation (Beltran et al., 2011; Dardenne et al., 2016; Lee et al., 2016). The overall mutational spectrum of NEPC has been reported to be similar to that of adenocarcinomas (Beltran et al., 2016; Davies et al., 2020). The lack of unique genomic alterations in NEPC as compared to adenocarcinomas imply an important role of epigenetic mechanisms (Davies et al., 2020) Studies suggest that epigenetic mechanisms, including DNA methylation and histone methylation alongwith transcriptional regulation play a huge role in the evolution of NEPC states. It is recognized that as tumors progress towards NEPC states, there is an increase in activity of stem-cell associated transcriptional programs (Smith et al., 2015; Lee et al., 2018; Smith et al., 2018). Recent years have seen a spurt in research delineating the epigenetic and transcriptional mechanisms underlying emergence of PCa NED states *via* stem-like states. In this article, we review the role of various transcription factors (TF) and chromatin modifiers that play a role in lineage reprogramming of prostate adenocarcinomas to NEPC under the selective pressure of various AR-targeted therapies (Figure 1). As studies shed light on the mechanistic basis of neuroendocrine differentiation in PCa, it is being recognized that these states are complex and heterogeneous. Based on the expression of TFs, the heterogeneity underlying these states is being dissected and has led to stratification of NEPC tumors. We highlight this stratification in this article. With the increased understanding, novel drugs/therapies are being tested in NEPC models. Here we review the current state of these therapies.

## 3 Androgen receptor: Still active in neuroendocrine prostate cancer?

It has been recognized that AR activity is varied in metastatic CRPC and can stratify distinct phenotypes (Labrecque et al., 2019). Five different molecular phenotypes have been reported: 1) AR high CRPC (ARPC); 2) AR low CRPC (ARLPC); 3) amphicrine PC (AMPC) that comprises of tumor cells co-expressing AR and NE gene programs; 4) SCNC that comprises of tumor cells expressing NE genes and negative for AR expression; 5) double negative PCa (DNPC) that is AR and NE double null (Labrecque et al., 2019). In SCNC, canonical AR signaling is typically lost/decreased (Beltran et al., 2016) though clinical studies have described SCNC subsets with retained AR expression and activity (Aggarwal et al., 2018). Though end state SCNC lack AR, more than 50% of treatment resistant PCa with NE features retain nuclear AR without activation of canonical AR signaling (Aggarwal et al., 2018; Labrecque et al., 2019; Alumkal et al., 2020). A recent study showed elegantly that AR cistrome undergoes alteration upon lineage reprogramming as a result of androgen deprivation (Davies et al., 2021). They reported that AR activity was maintained as prostate tumors adopt alternative NE lineage, with changes in chromatin architecture guiding AR transcriptional rerouting. AR cooperates with EZH2 to regulate lineage plasticity, with AR and EZH2 co-occupying the reprogrammed AR cistrome to transcriptionally modulate stem cell and neuronal gene networks (Davies et al., 2021) (Figure 1). In another study, AR has been shown to physically interact with Kruppel-like factor (KLF5) on chromatin sites upon endocrine therapy for CRPC. KLF5 is induced upon therapy in CRPC patients, binding to chromatin sites with AR co-occupying chromatin sites that drive opposing transcriptional programs. KLF5 is a stem cell transcription factor that promotes basal epithelial cell phenotypes concomitant with anchorage-independent growth and increased cell migration (Che et al., 2021). Furthermore, AR activity can be directly regulated by epigenetic modifiers such as histone deacetylase SIRT1 (Davies et al., 2020) that deacetylates AR and thereby prevents association with p300. SIRT1 is upregulated in NEPC and increased SIRT1 expression induces PCa NED by activating the Akt pathway (Ruan et al., 2018). In addition, the interaction between Akt and SIRT1 is independent of N-Myc and can drive NEPC development even when N-Myc is blocked (Ruan et al., 2018). Also, AR can directly recruit histone modifiers that influence chromatin states and gene expression (Davies et al., 2020). LSD1, H3-K4 demethylase is an important AR regulator that causes suppression of canonical AR transcriptional activity (Davies et al., 2020). LSD1 undergoes epigenetic reprogramming in CRPC that activates a subset of cell cycle genes, including CENPE, a centromere binding protein and mitotic kinesin. LSD1/AR binding and transactivation get reprogrammed by RB1 loss (Liang et al., 2017; Davies et al., 2020). These studies point to the crucial role of AR in promoting lineage reprogramming associated with PCa NE states.

## 4 EZH2: A master regulator of neuroendocrine prostate cancer reprogramming

Enhancer of zeste homolog 2 (EZH2), the catalytic subunit of Polycomb repressive complex 2 (PRC2), has been reported to be overexpressed in NEPC (11–13). EZH2 mediates the deposition of repressive histone modification, trimethylation of histone H3 at lysine 27 (H3K27m3) that represses lineage-specifying factors, thereby maintaining pluripotency (Lee et al., 2006) (Figure 1). It has been shown that EZH2 cooperates with oncogenic N-Myc to drive transcriptional programs leading to the evolution of PCa NE states (Dardenne et al., 2016). EZH2 is required for maintenance of bivalent chromatin states (with repressive as well as activating histone modifications) at N-Myc bound neuronal lineage-associated gene promoters. EZH2 knockdown leads to de-enrichment of neuronal-associated pathways in NEPC organoids (Berger et al., 2019). EZH2 depresses the TF SOX2 as a consequence of the functional loss of *RB1* (Kareta et al., 2015; Ku et al., 2017). An interesting study shows the role of EZH2 in repressing microRNA, miR-708 *via* binding to its promoter region (Shan et al., 2019). EZH2 is activated by transcription factor 4 (TCF4), a key component of Wnt/β-catenin signaling. EZH2-mediated repression of miR-708 leads to de-repression of neuronatin (Ryu et al., 2013) and stem cell factor CD44 (Saini et al., 2012). Furthermore, EZH2 activity is coupled to cAMP-response element binding protein (CREB) activation in prostate cancer (Zhang et al., 2018). Activation of CREB/EZH2 axis facilitates epigenetic repression of anti-angiogenic Thrombospondin 1 (TSP1), leading to angiogenesis and NE induction in PCa xenografts (Zhang et al., 2018). In agreement with a crucial role of EZH2 in PCa NED, EZH2 inhibitors are potential agents for NEPC treatment and have been shown to re-sensitize tumors to AR-signaling inhibitors in CRPC (Clermont et al., 2015; Beltran et al., 2016).

## 5 Other transcription factors

### 5.1 N-myc overexpression

*MYCN*, encoding the transcription factor, N-Myc is frequently amplified in neuroblastomas (Brodeur et al., 1984; Kohl et al., 1984), another type of NE tumor. *MYCN* is often overexpressed/amplified in NEPC and drives NE lineage reprogramming in conjunction with loss-of-function of *RB1* and *TP53* (Beltran et al., 2011). Forced expression of N-Myc in human prostate epithelial cells, along with Akt activation, led to aggressive tumors that histologically resemble NEPC (Dardenne et al., 2016). N-Myc cooperates with EZH2 to influence chromatin architecture and gene expression in NEPC. EZH2 inhibition could reverse the N-Myc-induced suppression of epithelial lineage genes in NEPC (Berger et al., 2019). AURKA stabilizes N-Myc (Beltran et al., 2011; Dardenne et al., 2016; Lee et al., 2016) and N-Myc co-binds with E2F1 to drive transcription of neuronal genes *CHGA*, *SYP* and *ENO2* (Liu et al., 2019). Furthermore, N-Myc is dependent on the BET family, in particular BRD4, to drive target gene expression (Henssen et al., 2016; Davies et al., 2020). BRD4 is responsible for maintenance of core stem cell genes such as NANOG and Oct4, in part, *via* its interaction with N-Myc. BRD4 binds to acetylated lysine residues in histone tails that leads to recruitment of positive transcription elongation factor b (p-TEFb). This recruitment of p-TEFb phosphorylates RNA polymerase II to activate gene expression (Davies et al., 2020). This interplay of TFs highlight that NEPC is driven by concerted action of an array of TFs that act in conjunction with chromatin modifiers that promote gene expression programs driving NED.

### 5.2 Role of POU family of TFs in neuroendocrine prostate cancer

Prominent players in NEPC are the lineage determinant transcription factors that drive neuroendocrine pathways (Andersen and Rosenfeld, 2001), amongst them are the neural POU domain transcription factors. POU (Pit-Oct-Unc) -domain/Oct proteins are a set of reprogramming TFs with important roles in neurogenesis. These TFs are critical regulators of gene expression programs determining cellular identities (Ishii et al., 2014; Jerabek et al., 2014; Chang et al., 2017). There are six classes of these TFs, with Class III POU genes (*POU3F1/OCT6, POU3F2/BRN2, POU3F3/BRN1, POU3F4/BRN4*) considered to be crucial for neurogenesis (Andersen and Rosenfeld, 2001). Out of these, following POU domain proteins have been implicated in NEPC.

#### 5.2.1 BRN2

BRN2 is an important driver of PCa NE states that is AR repressed and drives SOX2 expression (Figure 1) (Bishop et al., 2017). BRN2 and SOX2 act coordinately on chromatin to activate the expression of neuronal genes (Bishop et al., 2017). Clinically BRN2 expression has been observed to be elevated in NEPC (Bishop et al., 2017) and enzalutamide-resistant tumors (Fenner, 2017). Furthermore, BRN2 is known to synergistically upregulate several other transcription factors including SOX2, ASCL1 and PEG10 resulting in epigenetic modifications leading to NEPC. For this, BRN2 binds to the promoter region of SOX2 thereby promoting the expression of epigenetic modulators such as LSD1 and EZH2 (Kaarijarvi et al., 2021). A recent study identified MUC1-C protein that induces BRN2 expression *via* MYC activation (Yasumizu et al., 2020), further implicating it to another pathway for NEPC development.

#### 5.2.2 BRN4

Our lab demonstrated that BRN4 is over expressed in NEPC clinical samples and PDX models. We found that BRN4 interplays with BRN2 and regulates SOX2 expression (Figure 1) (Bhagirath et al., 2019). We further delineated a hitherto unknown mechanism by which during neuroendocrine differentiation, *BRN4* mRNA and protein is actively released in PCa cell exosomes/extracellular vesicles (EVs) upon NED alongwith BRN2. We showed that EV-mediated release of these TFs drive PCa NED (Bhagirath et al., 2019).

### 5.3 ONECUT2

ONECUT transcription factor family is involved in tumorigenesis. While ONECUT1 is associated with liver development and differentiation, ONECUT2 regulates cell proliferation, migration, adhesion and differentiation (Choi et al., 2022). Elevated expression levels of ONECUT2 have been reported in various cancers, particularly in NEPC (Choi et al., 2022). Therefore, ONECUT2 overexpression marks the onset of cancer progression and metastasis. Studies indicate ONECUT2 induces NED *via* suppressing AR signaling. Previously, interaction between AR receptors and ONECUT2 was speculated due to their coexistence (Rotinen et al., 2018; Guo et al., 2019). In CRPC, expression of AR and ONECUT2 are negatively correlated. The binding of ONECUT2 to AR has been studied *via* chromatin immunoprecipitation (ChIP) to further prove that ONECUT2 may be a direct repressor of AR signaling, thereby promoting NEPC (Rotinen et al., 2018). Hence, ONECUT2 could be a possible master regulator of neuroendocrine tumors (Figure 1). ONECUT2 is in network with other transcription factors including POU domain class 5 transcription factor 1, paired box protein (Pax-5), AR and EZH2 (Chang et al., 2017).

FOXA1 (Forkhead box A1) is known to be essential for the survival of normal prostate and prostate cancer tissue by maintaining AR signaling (Chang et al., 2017). The endogenous ONECUT2 binds to the promoter region of FOXA1 repressing its expression facilitating NED (Chang et al., 2017). A similar inverse correlation is observed between the expression of ONECUT2 and REST, another master repressor of neuronal differentiation (Ooi and Wood, 2007) (Lapuk et al., 2012). Apart from interaction with other master TFs, ONCECUT2 is involved in hypoxia signaling pathway promoting NED. Essentially, ONECUT2 activates SMAD3, that regulates hypoxia *via* targeting HIF1α chromatin binding, thus suppressing androgen signaling and facilitating NED (Guo et al., 2019).

### 5.4 SOX2

The SOX (Sry homology box) family of protein comprises of 20 individual proteins that are essentially defined by a conserved DNA Binding element- HMG (Kamachi and Kondoh, 2013). SOX proteins are classified into different subgroups on the basis of the HMG residue conservation. SOX2 belongs to the SOXB1 group along with SOX1 and SOX3 attributing to reprograming capacity, cell pluripotency, self-maintenance and stem cell maintenance (Zhang and Cui, 2014). In normal prostate, endogenous expression of SOX2 is found in partial basal epithelial cells. The SOX2 positive cells were positively correlated with Gleason score and metastases (Kregel et al., 2013). SOX2 expression is repressed by AR signaling. In concert with this, SOX2 repress the expression of prostate adenocarcinoma and synergistically with other factors, elevates neuroendocrine genes (Figure 1). However, this does not hamper the inhibitory effects of enzalutamide (Metz et al., 2020). SOX2 expression in PCa cells destabilizes the epithelial differentiation state and induce pluripotency genes, thereby driving neuroendocrine differentiation. Therefore, SOX2 expression is significantly higher in CRPC tumors with NE morphology than in adenocarcinoma (de Wet et al., 2022b). Furthermore, SOX2 is known to drive lineage plasticity in *RB1-* and *TP53*-deficient cancer cells (Mu et al., 2017). SOX2 expression promotes NED *via* enhancing the expression and enzymatic activity of demethylase LSD1, which is attributed to the global hypomethylation of histone H3 (Li et al., 2020). This leads to an altered epigenetic landscape of histone methylation, promoting lineage plasticity (Metz et al., 2020). Recent studies speculate that SOX2-mediated accelerated metastasis is possibly *via* enhanced metabolic activity. It has been reported that SOX2 expression confers high glycolysis and glycolytic capacity to PCa cells (de Wet et al., 2022a).

Hypoxia is often associated with later stage malignant prostate cancer. HIF proteins are crucial in the cellular responses to hypoxia. (Lin et al., 2011). An interplay between HIF-2α and SOX2 in PCa cells has been elucidated that suggest that lineage plasticity and stemness sets in to adapt in the hypoxic microenvironment. This corroborates the high expression of SOX2 and HIF-2α in CRPC and NEPC patient samples (Bae et al., 2016). A recent study demonstrates that TMPRSS4 upregulates the master EMT drivers SLUG and TWIST1 followed by subsequent SOX2 induction leading to CSC activation (Lee et al., 2021).

### 5.5 ASCL1

Transcription factors controlling early neurogenesis are critical for PCa NED (Vasconcelos and Castro, 2014). ASCL1 positively regulate neural progenitor differentiation and there is a strong correlation between ASCL1 expression and acquisition of NE-like features (Narayanan et al., 2019). ASCL1 upregulation is an early event following AR signaling suppression (Nouruzi et al., 2022). Hence, ASCL1 is also regarded as an efficient marker for aggressive phenotype and malignant cancer progression (Vias et al., 2008). Androgen deprivation triggers ASCL1 expression in LNCaP cells, whereas introduction of synthetic androgen reduced ASCL1. This strongly suggests that ASCL1 is highly responsive to androgen and maybe regulated by the AR signaling axis (Fraser et al., 2019). A recent study shows that targeting ASCL1 switches NE lineage to luminal epithelial state (Nouruzi et al., 2022). ASCL1 is enriched in hyper-accessible regions and functions predominantly by disrupting the epigenetic landscape of cancer cells and plays a pivotal role in the early chromatin remodeling in driving PCa NED. Prolonged use of enzalutamide treatment makes the DNA binding region of ASCL1 to be hyper-accessible. It can directly regulate neuronal and stem cell programs (Nouruzi et al., 2022). ASCL1 transcriptionally regulate UHRF1 and EZH2 activity. UHRF1 binds to AMPK to stabilize PRC2 complex and enhance histone H3-27-trimethylation. Loss of ASCL1 inhibits EZH2 activity and chromatin remodeling. This switches neuroendocrine phenotype to luminal lineage (Nouruzi et al., 2022).

### 5.6 NEUROD1

NEUROD1 is a neuronal TF that is able to convert epithelial cells into neurons (Lee et al., 1995). Therapy-induced NEPC can be classified to subtypes on the basis of ASCL1 and NEUROD1 expression profile (Cejas et al., 2021). RNA sequence analysis of PCa tissues focusing on DNA accessibility revealed distinct clustering of groups exclusively expressing NEUROD1 along with ASCL1 and AR. ChIP seq analysis of PCa divulged high enrichment of NEUROD1, ASCL1 and NFIB, plausible for the maintenance of chromatin state. NEUROD1 is potentially abundant of EBF and LHX motifs corroborating the high expression of neurogenic TFs EBF and LHX. (Cejas et al., 2021). Moreover, a strong association between the expression of NEPC marker Chromogranin A and NEUROD1 has been reported (Cindolo et al., 2007). Recent single cell transcriptomics revealed that NEUROD1 functions by collaborating with MYC for the initial NE oncogenesis (Wang et al., 2022).

### 5.7 REST

RE-1 silencing transcription factor is a master regulator of differentiation that represses neuronal programs in non-neuronal cells. REST downregulation has been shown to be associated with NEPC (Flores-Morales et al., 2019). SRRM4 is involved in alternative splicing of REST that leads to loss of its repressor activity in NE tumors (Shimojo et al., 2013; Li et al., 2017). SRRM4 incorporates exon N3c into the REST transcript, leading to the expression of truncated REST protein (REST4) that lacks the C-terminal repressor domain and thereby leads to induction of neuronal gene expression. SRRM3 was reported to have overlapping functions with SRRM4 and mediate alternative splicing of REST to REST4 (Labrecque et al., 2021b).

## 6 Other chromatin modifiers

### 6.1 HP1 family

Heterochromatin protein family is evolutionarily conserved for maintaining and regulating heterochromatin packaging. HP1 protein family consists of HP1α, HP1β, and HP1γ. As evolution of NEPC is driven *via* epigenetic chromatin remodeling, the role of this protein family is speculated. It is established that all three isoforms show altered expression in PCa tissues as compared to normal prostate tissues (Shapiro et al., 2008). HP1β serves as an AR coactivator, has a crucial role in transactivation of AR signaling, thereby promoting PCa cell proliferation (Shiota et al., 2010). HP1α has been implicated in NEPC (Ci et al., 2018). Expression of HP1α is elevated in NE PDX models and clinical samples. Its knockdown in NEPC cell line inhibits proliferation and induce apoptosis. Conversely, its ectopic expression significantly promotes NE transdifferentiation in adenocarcinoma cells subjected to androgen deprivation treatment. Mechanistically, HP1α reduces expression of AR and REST, enriching the repressive trimethylated histone H3 at Lys9 mark on their gene promoters (Ci et al., 2018).

### 6.2 SWI/SNF

SWI/SNF, also known as BAF (BRG1/Brahma Associated Factor), belongs to ATP-dependent chromatin remodeling complexes and is composed of 11–15 protein subunits (Kadoch and Crabtree, 2015). These are positive regulators of chromatin assembly and SWI/SNF alteration is critical in cancer development (Kadoch et al., 2013; Kadoch and Crabtree, 2015). Recent studies suggest the involvement of SWI/SNF in PCa trans-differentiation (Figure 1) in association with other lineage specific partners including NKX2.1, CHD4, MTA1 and VGF (Cyrta et al., 2020). Analysis of clinical samples identified an elevated level of SWI/SNF in CRPC-NE. Furthermore, SMARCA4 (BRG1) that has a pleiotropic role in genomic and/or in epigenetic modeling is high in aggressive PCa (Cyrta et al., 2020).

MUC1-C is an oncogenic protein that is predicted to have role in lineage plasticity. Studies show that MUC1-C directly binds to transcription factor E2F1. This axis elicits the expression of SWI/SNF complex components including BRG1, ARIDIA, BAF60a, BAF155 and BAF170 in CRPC and NEPC (Hagiwara et al., 2021). Moreover, MUC1 triggered significantly higher expression of E2F1 and eBAF in NEPC than in CRPC. MUC1 formed nuclear complex with BAF to activate cancer stem cells and pluripotency gene networking involving NOTCH1 and NANOG (Hagiwara et al., 2021).

### 6.3 BRD4

The Bromo domain and Extra terminal (BET) family of protein include BRD2, BRD3, BRD4 and BRDt. BRD4 consists of 2 bromo domains (BD1 and BD2) (Devaiah et al., 2016). These domains mediate the binding of BRD4 to chromatin *via* histones H3 and H4. BRD4 is known to play versatile roles as transcription factor, nucelators of superenhancers and kinase involved in transcription (Devaiah et al., 2016). BRD4 regulates cell migration across all CRPC models. It coregulates gene transcription to control cell migration and invasion through a large scaffolding protein, AHNAK. Furthermore MZ1, a small molecule BET inhibitor selectively degrades BRD4, inhibiting metastatic capacity of CRPC cell lines (Shafran et al., 2019). BRD4 also regulates expression of EMT genes SNAI1 and SNAI2 (Shafran et al., 2021). BRD4 is an oncogenic protein that cooperates with the transcription factor E2F1 to activate NE lineage plasticity (Kim et al., 2021). BRD4 has been getting considerable attention as potential anti-cancer drug target. Popular small molecule BRD4 inhibitors are JQ1 and iBET that show satisfactory result in managing advanced prostate cancer. Nevertheless, resistance to BET inhibitors has been reported in pre-clinical models (Fong et al., 2015). Although the molecular process behind this acquired resistance remain elusive, there is a study that links SPOP mutation as part of the mechanism. E3 ubiquitin ligase cullin 3 ^SPOP^ marks BRD4 for ubiquitin mediated degradation. However, SPOP mutants hamper the degradation causing an accumulation of BRD4 in PCa cells. This agrees with the inefficacy of BET inhibitors observed in SPOP mutant PCa cells (Dai et al., 2017). Proteolysis Targeting Chimera (PROTAC) technology-based BET degraders is shown to be more effective than small molecule BET inhibitors (Raina et al., 2016).

## 7 Crucial role of TFs in neuroendocrine prostate cancer: Stratification of neuroendocrine prostate cancer based on TFs

The cellular heterogeneity of prostate tumors progressing from AR-positive adenocarcinoma to AR negative NEPC allows stratification of this disease into various subtypes. Recent studies show that evolution to NE states involve an inherent hierarchical TF network. Single cell studies on CRPC and NEPC has shown that NEPC is driven by constitutive regulation of pioneering transcription factors ASCL1 and FOXA1 alongwith selective regulation of NKX2-2 or POUF3F2 and SOX2. This expression profile could categorize NEPC into subtypes based on transcriptomic mechanisms with NE1 expressing NKX2-2 and NE2 subtype expressing POU3F2 and SOX2 (Wang et al., 2022).

Cejas et al. (2021) reported that NEPC can be stratified on the basis of expression of the neuronal transcription factors ASCL1 and NEUROD1 , like SCLC (Rudin et al., 2019). ChIP-Seq analyses of these two TFs in NEPC models showed that there are thousands of highly conserved binding sites with both overlapping and differential binding sites. *De novo* motif analyses of these binding sites showed that these binding sites possess the respective consensus motifs for ASCL1 and NEUROD1. Further, ASCL1 binding motif was found to be enriched with motif for NKX2 (Cejas et al., 2021), in keeping with reports that NKX2-1 TF is 16-fold highly expressed in the ASCL1 subtype of NE tumors (Borromeo et al., 2016). NEUROD1 binding motif was enriched for EBF and LHX motifs corresponding to the higher expression of TFs EBF and LHX in NEUROD1 subtype. Gene set enrichment analyses to identify pathways represented in ASCL1 and NEUROD1 subtypes showed that ASCL1 subtype was enriched in GO pathways of response to cytokines while NEUROD1 subtype showed enrichment for brain development pathways (Cejas et al., 2021). While the NE PDX models show a mutually exclusive expression of ASCL1 and NEUROD1, human NE tumors were found to exhibit a complex tumor structure with subtypes co-existing as separate sub-populations within the same tumor (Cejas et al., 2021).

Labreque et al. stratified NEPC based on expression of RNA splicing factors serine/arginine repetitive matrix protein 4 (SRRM4) and SRRM3 (Labrecque et al., 2021b) that control alternative splicing of REST. REST is a repressor of neuronal genes that is downregulated in NEPC (Flores-Morales et al., 2019). SRRM4 is involved in alternative splicing of REST wherein it incorporates exon N3c into the REST transcript leading to the expression of truncated REST protein (REST4) that lacks the C-terminal repressor domain that leads to loss of its repressor activity and induction of neuronal genes (Shimojo et al., 2013; Li et al., 2017). SRRM3 have overlapping functions with SRRM4 and mediates alternative splicing of REST to REST4 (Labrecque et al., 2021b). Based on SRRM4 and SRRM3 expression, three molecular subtypes of SCNC were characterized that were found to be progressively neuronal: 1) SCNC-1: These tumors express SRRM3, driving alternative splicing of REST to REST4. These SCNC tumors co-express ASCL1; 2) SCNC-2: This subtype of SCNC express SRRM3 and SRRM4 alongwith neuronal MYCN, NEUROD1 and NEUROD4 expression. These tumors express significantly upregulated transcription factors such as ZNF536, ISL1, PAX6 and MYT1L; 3) SCNC-3: A subtype of SCNC that express SRRM3, SRRM4 accompanied with MYCN and NEUROD6. The neural transcription factors upregulated in SCNC-3 tumors included BARHL1, LHX3, UNCX, POU3F3, POU4F2, POU4F3 and ATOH1. This study suggests that the biology of SCNC-1 is strikingly different from that of SCNC-2 and -3 and is expected to respond differently to therapies. Since SCNC-1 is N-Myc null, it is not expected to respond to AURKA inhibitor Alisertib that acts by disrupting AURKA-N-Myc interaction (Beltran et al., 2019). Further, SCNC-2 tumors could represent tumors transitioning to SCNC-3 (Ku et al., 2017). These studies highlight the heterogeneity of NEPC and suggest that therapeutic strategies need to be designed accordingly.

## 8 Targeting the epigenome as a therapeutic strategy for neuroendocrine prostate cancer

Currently, the standard of care for NEPC is platinum based drugs that show a short duration of response and are administered to all NEPC patients (Aparicio et al., 2013). With an increased understanding of the epigenetic basis of NEPC, therapeutic startegies targeting epigenetic mechanisms are being tested for targeting NEPC effectively (Table 1). Table 2 lists the clinical trials in NEPC focused on TFs, chromatin modifiers and other proteins. EZH2 inhibitors are potential agents for NEPC treatment and have shown promise in pre-clinical studies in reversing the NE phenotype established by N-Myc (Dardenne et al., 2016; Berger et al., 2019) or *RB1*/*TP53* loss. EZH2 inhibitors PF-06821,497 and CPI-1205 are currently being tested in clinical trials in CRPC (NCT03460977 and NCT03480646, respectively) (Davies et al., 2020). CPI-1205 is being tested in combination with enzalutamide or abiraterone/prednisone and has shown promising activity (Davies et al., 2020). Considering preclinical evidence for EZH2 inhibition leading to inhibition of NE differentiation, EZH2 is an important target for NEPC (Ku et al., 2017). These inhibitors have been shown to re-sensitize tumors to AR-signaling inhibitors in CRPC (Clermont et al., 2015; Beltran et al., 2016).

**TABLE 1:** Preclinical evaluation of therapeutic strategies targeting TFs and chromatin modifiers in NEPC.

Therapeutic target	Drug	Mechanism of action/effect
EZH2 inhibitors	Tazemetostat, GSK126	Resensitize tumor to AR-signaling inhibitors (Beltran et al., 2016; Clermont et al., 2015)
AURKA inhibitors	Alisertib	Inhibits interaction between NMYC and Aurora-A, suppressing tumor growth (Beltran et al., 2019)
ONECUT2 inhibitor	CSRM617	Reduce proliferation and tumor volume by inducing apoptosis (Choi et al., 2022)
BET inhibitors	MZ1	Degrades BRD4 and controls CRPC metastasis (Shafran et al., 2019)
	JQ1, iBET	Controls CRPC metastasis ((Kim et al., 2021)

**TABLE 2:** Clinical Trials in NEPC focused on TFs, chromatin modifiers and other proteins.

Clinical trial number	Drug	Efficacy
NCT05413421 (Phase I/Ib)	PRC2 inhibitor, ORIC-944	Not reported (ongoing)
NCT01799278 (Phase II)	AURKA inhibitor, Alisertib	A subset of patients with advanced prostate cancer and molecular features supporting Aurora-A and N-myc activation achieved significant clinical benefit (Beltran et al., 2019)
NCT04702737 (Phase 1b)	DLL3 bispecific T Cell engager, AMG 757	Not reported (ongoing)
NCT05268666 (Phase I/II)	LSD1/HDAC6 inhibitor, JBI-802	Not reported (ongoing)
NCT02711956 (Phase Ib/IIa)	BETi, ZEN-3694	ZEN-3694 plus enzalutamide demonstrated acceptable tolerability and potential efficacy in patients with ASI-resistant mCRPC (Aggarwal et al., 2020)

Targeting other epigenetic regulators such as BET family are also being investigated in advanced CRPC (Asangani et al., 2014) and may have rationale in NEPC (Beltran and Demichelis, 2021; Conteduca et al., 2021). BET inhibition using compound JQ1 disrupts the recruitment of AR to target gene sites (Asangani et al., 2014) BET inhibition blocks E2F1/BRD4-regulated program and decreases growth of NEPC tumor models. Further, a subset of t-NEPC patient tumors with high activity of this program showed decreased tumor growth in a BETi clinical trial (Kim et al., 2021). Aggarwal et al. (2020) tested the activity of BETi ZEN-3694 in a phase IIb/IIa study in metastatic CRPC patients with resistance to AR signaling pathway inhibitors and reported acceptable tolerability and potential efficacy in these patients. Importantly, tumors with low baseline AR activity appeared to derive greater benefit than patients with high baseline AR activity (Aggarwal et al., 2020).

Disruption of the molecular interaction between AURKA and N-Myc by Alisertib (MLN8237) has been examined as a therapeutic modality for NEPC (Beltran et al., 2019). A phase-II clinical trial of Alisertib reported modest clinical benefit of Alisertib in NEPC patients while a second phase I/II trial in combination with abiraterone in CRPC-NE had to be terminated due to toxicity and non-significant clinical benefit (Davies et al., 2020).

Targeting of TFs that mediate transcriptional changes during lineage reprogramming such as BRN2, BRN4, MYCN, ASCL1, FOXA1 and ONECUT2 can be exploited as a therpeutic strategy for NEPC. However, TFs are challenging to target directly (Beltran and Demichelis, 2021). BRN2 inhibition *via* siRNA or CRISPR/Cas9 or small molecule inhibitor was found to reduce proliferation in NEPC cell lines (Daksh et al., 2019). CSRM617 is a small molecular ONECUT2 inhibitor developed on the basis of a three-dimensional model of ONECUT2. This is found effective in reducing proliferation and tumor volume by inducing apoptosis of cancer cells with high ONECUT2 expression (Cully, 2018).

With the recent studies highlighting the enormous heterogeneity of PCa NE states and stratification based on expression of distinct TFs, the transcriptional programs of these subtypes are likely distinct. This emphasizes the requirement for specific therapeutic strategies focused on targeting distinct master regulators of each subtype for effective therapy. Considering the upregulation of Delta-like protein 3 (DLL3) in NEPC cases as compared to CRPC-adenocarcinomas, a humanized DLL3 antibody has been exploited for therapeutic targeting of NEPC (2019, Puca et al., 2019; Thoma, 2019). This therapeutic modality is likely to be effective in the ASCL1 subtype of NEPC (Cejas et al., 2021). AURKA inhibition may be more efficacious in the NEUROD1 subtype, similar to the case in SCLC (Mollaoglu et al., 2017). Therapies targeting CEACAM5 such as carcinoembryonic antigen-related cell adhesion molecule CEACAM5 antibody-drug conjugate (DeLucia et al., 2021) is likely to be efficacious in ASCL1 subtype of NEPC since this subtype was reported to be enriched in CEACAM 1,5,6,7 (Cejas et al., 2021). Effective therapeutic strategies need to be designed that would target the different subpopulations to avoid emergence of outgrowth of the resistant subpopulations (Cejas et al., 2021). There is a need to develop biomarker-driven treatment strategies for NEPC. Furthermore, owing to inter and intra-patient heterogeneity, it will be critical to gain better insights into the underlying biology of these tumors and then design combinatorial therapeutic strategies targeting factors important in specific tumor subsets (Labrecque et al., 2021a). Though progress is being made in this direction, we still have a long way to go before these therapies can be effectively implemented in the clinic.

## 9 Conclusion

In conclusion, transcription factors and chromatin modifiers play an integral role in driving neuroendocrine trans-differentiation in prostate cancer. Pioneer transcription factors such as ASCL1, FOXA1 and SOX2 can engage on their respective target sites on nucleosomal DNA and initiate transcriptional regulation at sites of closed chromatin. Further, interplay between transcription factors such as AR, FOXA1, BRN2 and BRN4 and epigenetic modifiers such as EZH2 drive chromatin states underlying lineage reprogramming in PCa. The complexities underlying emergence and maintenance of PCa NE states is being recognized and deciphered. With an increased understanding of the temporal and spatial interplay of transcription factors and their associated gene expression programs in PCa NED, better therapeutic strategies can be designed for targeting NEPC. Though considerable progress have been made in this direction in last few years, we are still far from understanding the complexy regulatory interplay. Further, it is important to unravel the interplay between these TFs, epigenetic modifiers and tumor microenvironment to gain a holistic understanding of the disease that would be required for devising effective therapeutic modalities.

## References

[B1] Aggarwal R. Huang J. Alumkal J. J. Zhang L. Feng F. Y. Thomas G. V. (2018). Clinical and genomic characterization of treatment-emergent small-cell neuroendocrine prostate cancer: A multi-institutional prospective study. J. Clin. Oncol. 36, 2492–2503. 10.1200/JCO.2017.77.6880 29985747 PMC6366813

[B2] Aggarwal R. R. Schweizer M. T. Nanus D. M. Pantuck A. J. Heath E. I. Campeau E. (2020). A phase ib/IIa study of the pan-BET inhibitor ZEN-3694 in combination with enzalutamide in patients with metastatic castration-resistant prostate cancer. Clin. Cancer Res. 26, 5338–5347. 10.1158/1078-0432.CCR-20-1707 32694156 PMC7572827

[B3] Aggarwal R. R. Small E. J. (2014). Small-cell/neuroendocrine prostate cancer: A growing threat? Oncol. Willist. Park) 28, 838–840. 25323608

[B4] Aggarwal R. Zhang T. Small E. J. Armstrong A. J. (2014). Neuroendocrine prostate cancer: Subtypes, biology, and clinical outcomes. J. Natl. Compr. Canc Netw. 12, 719–726. 10.6004/jnccn.2014.0073 24812138

[B5] Alumkal J. J. Sun D. Lu E. Beer T. M. Thomas G. V. Latour E. (2020). Transcriptional profiling identifies an androgen receptor activity-low, stemness program associated with enzalutamide resistance. Proc. Natl. Acad. Sci. U. S. A. 117, 12315–12323. 10.1073/pnas.1922207117 32424106 PMC7275746

[B6] Andersen B. Rosenfeld M. G. (2001). POU domain factors in the neuroendocrine system: Lessons from developmental biology provide insights into human disease. Endocr. Rev. 22, 2–35. 10.1210/edrv.22.1.0421 11159814

[B7] Aparicio A. M. Harzstark A. L. Corn P. G. Wen S. Araujo J. C. Tu S. M. (2013). Platinum-based chemotherapy for variant castrate-resistant prostate cancer. Clin. Cancer Res. 19, 3621–3630. 10.1158/1078-0432.CCR-12-3791 23649003 PMC3699964

[B8] Asangani I. A. Dommeti V. L. Wang X. Malik R. Cieslik M. Yang R. (2014). Therapeutic targeting of BET bromodomain proteins in castration-resistant prostate cancer. Nature 510, 278–282. 10.1038/nature13229 24759320 PMC4075966

[B9] Bae K. M. Dai Y. Vieweg J. Siemann D. W. (2016). Hypoxia regulates SOX2 expression to promote prostate cancer cell invasion and sphere formation. Am. J. Cancer Res. 6, 1078–1088. 27294000 PMC4889721

[B10] Balanis N. G. Sheu K. M. Esedebe F. N. Patel S. J. Smith B. A. Park J. W. (2019). Pan-cancer convergence to a small-cell neuroendocrine phenotype that shares susceptibilities with hematological malignancies. Cancer Cell 36, 17–34. 10.1016/j.ccell.2019.06.005 31287989 PMC6703903

[B11] Beltran H. Demichelis F. (2021). Therapy considerations in neuroendocrine prostate cancer: What next? Endocr. Relat. Cancer 28, T67–T78. 10.1530/ERC-21-0140 34111024 PMC8289743

[B12] Beltran H. Oromendia C. Danila D. C. Montgomery B. Hoimes C. Szmulewitz R. Z. (2019). A phase II trial of the Aurora kinase A inhibitor Alisertib for patients with castration-resistant and neuroendocrine prostate cancer: Efficacy and biomarkers. Clin. Cancer Res. 25, 43–51. 10.1158/1078-0432.CCR-18-1912 30232224 PMC6320304

[B13] Beltran H. Prandi D. Mosquera J. M. Benelli M. Puca L. Cyrta J. (2016). Divergent clonal evolution of castration-resistant neuroendocrine prostate cancer. Nat. Med. 22, 298–305. 10.1038/nm.4045 26855148 PMC4777652

[B14] Beltran H. Rickman D. S. Park K. Chae S. S. Sboner A. Macdonald T. Y. (2011). Molecular characterization of neuroendocrine prostate cancer and identification of new drug targets. Cancer Discov. 1, 487–495. 10.1158/2159-8290.CD-11-0130 22389870 PMC3290518

[B15] Berger A. Brady N. J. Bareja R. Robinson B. Conteduca V. Augello M. A. (2019). N-Myc-mediated epigenetic reprogramming drives lineage plasticity in advanced prostate cancer. J. Clin. Invest. 129, 3924–3940. 10.1172/JCI127961 31260412 PMC6715370

[B16] Bhagirath D. Yang T. L. Tabatabai Z. L. Majid S. Dahiya R. Tanaka Y. (2019). BRN4 is a novel driver of neuroendocrine differentiation in castration-resistant prostate cancer and is selectively released in extracellular vesicles with BRN2. Clin. Cancer Res. 25, 6532–6545. 10.1158/1078-0432.CCR-19-0498 31371344 PMC6825556

[B17] Bishop J. L. Thaper D. Vahid S. Davies A. Ketola K. Kuruma H. (2017). The master neural transcription factor BRN2 is an androgen receptor-suppressed driver of neuroendocrine differentiation in prostate cancer. Cancer Discov. 7, 54–71. 10.1158/2159-8290.CD-15-1263 27784708

[B18] Borromeo M. D. Savage T. K. Kollipara R. K. He M. Augustyn A. Osborne J. K. (2016). ASCL1 and NEUROD1 reveal heterogeneity in pulmonary neuroendocrine tumors and regulate distinct genetic programs. Cell Rep. 16, 1259–1272. 10.1016/j.celrep.2016.06.081 27452466 PMC4972690

[B19] Brodeur G. M. Seeger R. C. Schwab M. Varmus H. E. Bishop J. M. (1984). Amplification of N-myc in untreated human neuroblastomas correlates with advanced disease stage. Science 224, 1121–1124. 10.1126/science.6719137 6719137

[B20] Cejas P. Xie Y. Font-Tello A. Lim K. Syamala S. Qiu X. (2021). Subtype heterogeneity and epigenetic convergence in neuroendocrine prostate cancer. Nat. Commun. 12, 5775. 10.1038/s41467-021-26042-z 34599169 PMC8486778

[B21] Chang Y. K. Srivastava Y. Hu C. Joyce A. Yang X. Zuo Z. (2017). Quantitative profiling of selective Sox/POU pairing on hundreds of sequences in parallel by Coop-seq. Nucleic Acids Res. 45, 832–845. 10.1093/nar/gkw1198 27915232 PMC5314778

[B22] Che M. Chaturvedi A. Munro S. A. Pitzen S. P. Ling A. Zhang W. (2021). Opposing transcriptional programs of KLF5 and AR emerge during therapy for advanced prostate cancer. Nat. Commun. 12, 6377. 10.1038/s41467-021-26612-1 34737261 PMC8568894

[B23] Choi W. W. Boland J. L. Lin J. (2022). ONECUT2 as a key mediator of androgen receptor-independent cell growth and neuroendocrine differentiation in castration-resistant prostate cancer. Cancer Drug Resist 5, 165–170. 10.20517/cdr.2021.108 35582526 PMC8992592

[B24] Ci X. Hao J. Dong X. Choi S. Y. Xue H. Wu R. (2018). Heterochromatin protein 1α mediates development and aggressiveness of neuroendocrine prostate cancer. Cancer Res. 78, 2691–2704. 10.1158/0008-5472.CAN-17-3677 29487201

[B25] Cindolo L. Franco R. Cantile M. Schiavo G. Liguori G. Chiodini P. (2007). NeuroD1 expression in human prostate cancer: Can it contribute to neuroendocrine differentiation comprehension? Eur. Urol. 52, 1365–1373. 10.1016/j.eururo.2006.11.030 17126478

[B26] Clermont P. L. Lin D. Crea F. Wu R. Xue H. Wang Y. (2015). Polycomb-mediated silencing in neuroendocrine prostate cancer. Clin. Epigenetics 7, 40. 10.1186/s13148-015-0074-4 25859291 PMC4391120

[B27] Conteduca V. Hess J. Yamada Y. Ku S. Y. Beltran H. (2021). Epigenetics in prostate cancer: Clinical implications. Transl. Androl. Urol. 10, 3104–3116. 10.21037/tau-20-1339 34430414 PMC8350251

[B28] Culig Z. (2017). Molecular mechanisms of enzalutamide resistance in prostate cancer. Curr. Mol. Biol. Rep. 3, 230–235. 10.1007/s40610-017-0079-1 29214142 PMC5700216

[B29] Cully M. (2018). Anticancer drugs: Cutting down on prostate cancer metastases. Nat. Rev. Drug Discov. 18, 17. 10.1038/nrd.2018.225 30591730

[B30] Cyrta J. Augspach A. De Filippo M. R. Prandi D. Thienger P. Benelli M. (2020). Role of specialized composition of SWI/SNF complexes in prostate cancer lineage plasticity. Nat. Commun. 11, 5549. 10.1038/s41467-020-19328-1 33144576 PMC7642293

[B31] Dai X. Wang Z. Wei W. (2017). SPOP-mediated degradation of BRD4 dictates cellular sensitivity to BET inhibitors. Cell Cycle 16, 2326–2329. 10.1080/15384101.2017.1388973 29108467 PMC5788415

[B32] Daksh L. Thaper R. M. Shaghayegh N. O. U. R. U. Z. I. Sahil K. U. M. A. R. Soojin K. I. M. Sepideh V. A. H. I. D. (2019). First-in-field small molecule inhibitors targeting BRN2 as a therapeutic strategy for small cell prostate cancer. J. Clin. Oncol. 37, 260. 10.1200/jco.2019.37.7_suppl.260

[B33] Dardenne E. Beltran H. Benelli M. Gayvert K. Berger A. Puca L. (2016). N-myc induces an EZH2-mediated transcriptional program driving neuroendocrine prostate cancer. Cancer Cell 30, 563–577. 10.1016/j.ccell.2016.09.005 27728805 PMC5540451

[B34] Davies A. Nouruzi S. Ganguli D. Namekawa T. Thaper D. Linder S. (2021). An androgen receptor switch underlies lineage infidelity in treatment-resistant prostate cancer. Nat. Cell Biol. 23, 1023–1034. 10.1038/s41556-021-00743-5 34489572 PMC9012003

[B35] Davies A. Zoubeidi A. Selth L. A. (2020). The epigenetic and transcriptional landscape of neuroendocrine prostate cancer. Endocr. Relat. Cancer 27, R35–R50. 10.1530/ERC-19-0420 31804971

[B36] De Wet L. Williams A. Gillard M. Kregel S. Lamperis S. Gutgesell L. C. (2022a). Correction to: SOX2 mediates metabolic reprogramming of prostate cancer cells. Oncogene 41, 1234. 10.1038/s41388-022-02228-7 35145235

[B37] De Wet L. Williams A. Gillard M. Kregel S. Lamperis S. Gutgesell L. C. (2022b). SOX2 mediates metabolic reprogramming of prostate cancer cells. Oncogene 41, 1190–1202. 10.1038/s41388-021-02157-x 35067686 PMC8858874

[B39] Delucia D. C. Cardillo T. M. Ang L. Labrecque M. P. Zhang A. Hopkins J. E. (2021). Regulation of CEACAM5 and therapeutic efficacy of an anti-CEACAM5-SN38 antibody-drug conjugate in neuroendocrine prostate cancer. Clin. Cancer Res. 27, 759–774. 10.1158/1078-0432.CCR-20-3396 33199493 PMC7854497

[B40] Devaiah B. N. Gegonne A. Singer D. S. (2016). Bromodomain 4: A cellular Swiss army knife. J. Leukoc. Biol. 100, 679–686. 10.1189/jlb.2RI0616-250R 27450555 PMC5014741

[B41] Epstein J. I. Amin M. B. Beltran H. Lotan T. L. Mosquera J. M. Reuter V. E. (2014). Proposed morphologic classification of prostate cancer with neuroendocrine differentiation. Am. J. Surg. Pathol. 38, 756–767. 10.1097/PAS.0000000000000208 24705311 PMC4112087

[B42] Fenner A. (2017). Prostate cancer: BRN2 is a neuroendocrine driver. Nat. Rev. Urol. 14, 10. 10.1038/nrurol.2016.237 27843142

[B43] Flores-Morales A. Bergmann T. B. Lavallee C. Batth T. S. Lin D. Lerdrup M. (2019). Proteogenomic characterization of patient-derived xenografts highlights the role of REST in neuroendocrine differentiation of castration-resistant prostate cancer. Clin. Cancer Res. 25, 595–608. 10.1158/1078-0432.CCR-18-0729 30274982

[B44] Fong C. Y. Gilan O. Lam E. Y. Rubin A. F. Ftouni S. Tyler D. (2015). BET inhibitor resistance emerges from leukaemia stem cells. Nature 525, 538–542. 10.1038/nature14888 26367796 PMC6069604

[B45] Fraser J. A. Sutton J. E. Tazayoni S. Bruce I. Poole A. V. (2019). hASH1 nuclear localization persists in neuroendocrine transdifferentiated prostate cancer cells, even upon reintroduction of androgen. Sci. Rep. 9, 19076. 10.1038/s41598-019-55665-y 31836808 PMC6911083

[B46] Guo H. Ci X. Ahmed M. Hua J. T. Soares F. Lin D. (2019). ONECUT2 is a driver of neuroendocrine prostate cancer. Nat. Commun. 10, 278. 10.1038/s41467-018-08133-6 30655535 PMC6336817

[B47] Hagiwara M. Yasumizu Y. Yamashita N. Rajabi H. Fushimi A. Long M. D. (2021). MUC1-C activates the BAF (mSWI/SNF) complex in prostate cancer stem cells. Cancer Res. 81, 1111–1122. 10.1158/0008-5472.CAN-20-2588 33323379 PMC8026569

[B48] Henssen A. Althoff K. Odersky A. Beckers A. Koche R. Speleman F. (2016). Targeting MYCN-driven transcription by BET-bromodomain inhibition. Clin. Cancer Res. 22, 2470–2481. 10.1158/1078-0432.CCR-15-1449 26631615

[B49] Ishii J. Sato H. Yazawa T. Shishido-Hara Y. Hiramatsu C. Nakatani Y. (2014). Class III/IV POU transcription factors expressed in small cell lung cancer cells are involved in proneural/neuroendocrine differentiation. Pathol. Int. 64, 415–422. 10.1111/pin.12198 25243889

[B50] Jerabek S. Merino F. Scholer H. R. Cojocaru V. (2014). OCT4: Dynamic DNA binding pioneers stem cell pluripotency. Biochim. Biophys. Acta 1839, 138–154. 10.1016/j.bbagrm.2013.10.001 24145198

[B51] Kaarijarvi R. Kaljunen H. Ketola K. (2021). Molecular and Functional Links between Neurodevelopmental Processes and Treatment-Induced Neuroendocrine Plasticity in Prostate Cancer Progression, Cancers (Basel) 13, 692. 10.3390/cancers13040692 33572108 PMC7915380

[B52] Kadoch C. Crabtree G. R. (2015). Mammalian SWI/SNF chromatin remodeling complexes and cancer: Mechanistic insights gained from human genomics. Sci. Adv. 1, e1500447. 10.1126/sciadv.1500447 26601204 PMC4640607

[B53] Kadoch C. Hargreaves D. C. Hodges C. Elias L. Ho L. Ranish J. (2013). Proteomic and bioinformatic analysis of mammalian SWI/SNF complexes identifies extensive roles in human malignancy. Nat. Genet. 45, 592–601. 10.1038/ng.2628 23644491 PMC3667980

[B54] Kamachi Y. Kondoh H. (2013). Sox proteins: Regulators of cell fate specification and differentiation. Development 140, 4129–4144. 10.1242/dev.091793 24086078

[B55] Kareta M. S. Gorges L. L. Hafeez S. Benayoun B. A. Marro S. Zmoos A. F. (2015). Inhibition of pluripotency networks by the Rb tumor suppressor restricts reprogramming and tumorigenesis. Cell Stem Cell 16, 39–50. 10.1016/j.stem.2014.10.019 25467916 PMC4389904

[B56] Kim D. H. Sun D. Storck W. K. Welker Leng K. Jenkins C. Coleman D. J. (2021). BET bromodomain inhibition blocks an AR-repressed, E2F1-activated treatment-emergent neuroendocrine prostate cancer lineage plasticity program. Clin. Cancer Res. 27, 4923–4936. 10.1158/1078-0432.CCR-20-4968 34145028 PMC8416959

[B57] Klimstra D. S. Beltran H. Lilenbaum R. Bergsland E. (2015). The spectrum of neuroendocrine tumors: Histologic classification, unique features and areas of overlap. Am. Soc. Clin. Oncol. Educ. Book, 92–103. 10.14694/EdBook_AM.2015.35.92 25993147

[B58] Knudsen K. E. Scher H. I. (2009). Starving the addiction: New opportunities for durable suppression of AR signaling in prostate cancer. Clin. Cancer Res. 15, 4792–4798. 10.1158/1078-0432.CCR-08-2660 19638458 PMC2842118

[B59] Kohl N. E. Gee C. E. Alt F. W. (1984). Activated expression of the N-myc gene in human neuroblastomas and related tumors. Science 226, 1335–1337. 10.1126/science.6505694 6505694

[B60] Kregel S. Kiriluk K. J. Rosen A. M. Cai Y. Reyes E. E. Otto K. B. (2013). Sox2 is an androgen receptor-repressed gene that promotes castration-resistant prostate cancer. PLoS One 8, e53701. 10.1371/journal.pone.0053701 23326489 PMC3543364

[B61] Ku S. Y. Rosario S. Wang Y. Mu P. Seshadri M. Goodrich Z. W. (2017). Rb1 and Trp53 cooperate to suppress prostate cancer lineage plasticity, metastasis, and antiandrogen resistance. Science 355, 78–83. 10.1126/science.aah4199 28059767 PMC5367887

[B62] Labrecque M. P. Alumkal J. J. Coleman I. M. Nelson P. S. Morrissey C. (2021a). The heterogeneity of prostate cancers lacking AR activity will require diverse treatment approaches. Endocr. Relat. Cancer 28, T51–T66. 10.1530/ERC-21-0002 33792558 PMC8292199

[B63] Labrecque M. P. Brown L. G. Coleman I. M. Lakely B. Brady N. J. Lee J. K. (2021b). RNA splicing factors SRRM3 and SRRM4 distinguish molecular phenotypes of castration-resistant neuroendocrine prostate cancer. Cancer Res. 81, 4736–4750. 10.1158/0008-5472.CAN-21-0307 34312180 PMC8448969

[B64] Labrecque M. P. Coleman I. M. Brown L. G. True L. D. Kollath L. Lakely B. (2019). Molecular profiling stratifies diverse phenotypes of treatment-refractory metastatic castration-resistant prostate cancer. J. Clin. Invest. 129, 4492–4505. 10.1172/JCI128212 31361600 PMC6763249

[B65] Lapuk A. V. Wu C. Wyatt A. W. Mcpherson A. Mcconeghy B. J. Brahmbhatt S. (2012). From sequence to molecular pathology, and a mechanism driving the neuroendocrine phenotype in prostate cancer. J. Pathol. 227, 286–297. 10.1002/path.4047 22553170 PMC3659819

[B66] Lee A. R. Gan Y. Tang Y. Dong X. (2018). A novel mechanism of SRRM4 in promoting neuroendocrine prostate cancer development via a pluripotency gene network. EBioMedicine 35, 167–177. 10.1016/j.ebiom.2018.08.011 30100395 PMC6154886

[B67] Lee J. E. Hollenberg S. M. Snider L. Turner D. L. Lipnick N. Weintraub H. (1995). Conversion of Xenopus ectoderm into neurons by NeuroD, a basic helix-loop-helix protein. Science 268, 836–844. 10.1126/science.7754368 7754368

[B68] Lee J. K. Phillips J. W. Smith B. A. Park J. W. Stoyanova T. Mccaffrey E. F. (2016). N-myc drives neuroendocrine prostate cancer initiated from human prostate epithelial cells. Cancer Cell 29, 536–547. 10.1016/j.ccell.2016.03.001 27050099 PMC4829466

[B69] Lee T. I. Jenner R. G. Boyer L. A. Guenther M. G. Levine S. S. Kumar R. M. (2006). Control of developmental regulators by Polycomb in human embryonic stem cells. Cell 125, 301–313. 10.1016/j.cell.2006.02.043 16630818 PMC3773330

[B70] Lee Y. Yoon J. Ko D. Yu M. Lee S. Kim S. (2021). TMPRSS4 promotes cancer stem-like properties in prostate cancer cells through upregulation of SOX2 by SLUG and TWIST1. J. Exp. Clin. Cancer Res. 40, 372. 10.1186/s13046-021-02147-7 34809669 PMC8607621

[B71] Li H. Wang L. Li Z. Geng X. Li M. Tang Q. (2020). SOX2 has dual functions as a regulator in the progression of neuroendocrine prostate cancer. Lab. Invest. 100, 570–582. 10.1038/s41374-019-0343-5 31772313

[B72] Li Y. Donmez N. Sahinalp C. Xie N. Wang Y. Xue H. (2017). SRRM4 drives neuroendocrine transdifferentiation of prostate adenocarcinoma under androgen receptor pathway inhibition. Eur. Urol. 71, 68–78. 10.1016/j.eururo.2016.04.028 27180064

[B73] Liang Y. Ahmed M. Guo H. Soares F. Hua J. T. Gao S. (2017). LSD1-Mediated epigenetic reprogramming drives CENPE expression and prostate cancer progression. Cancer Res. 77, 5479–5490. 10.1158/0008-5472.CAN-17-0496 28916652

[B74] Lin Q. Cong X. Yun Z. (2011). Differential hypoxic regulation of hypoxia-inducible factors 1alpha and 2alpha. Mol. Cancer Res. 9, 757–765. 10.1158/1541-7786.MCR-11-0053 21571835 PMC3117969

[B75] Liu B. Li L. Yang G. Geng C. Luo Y. Wu W. (2019). PARP inhibition suppresses GR-MYCN-CDK5-RB1-E2F1 signaling and neuroendocrine differentiation in castration-resistant prostate cancer. Clin. Cancer Res. 25, 6839–6851. 10.1158/1078-0432.CCR-19-0317 31439587 PMC6858969

[B76] Lotan T. L. Gupta N. S. Wang W. Toubaji A. Haffner M. C. Chaux A. (2011). ERG gene rearrangements are common in prostatic small cell carcinomas. Mod. Pathol. 24, 820–828. 10.1038/modpathol.2011.7 21336263 PMC3484363

[B77] Maina P. K. Shao P. Liu Q. Fazli L. Tyler S. Nasir M. (2016). c-MYC drives histone demethylase PHF8 during neuroendocrine differentiation and in castration-resistant prostate cancer. Oncotarget 7, 75585–75602. 10.18632/oncotarget.12310 27689328 PMC5342763

[B78] Metz E. P. Wilder P. J. Dong J. Datta K. Rizzino A. (2020). Elevating SOX2 in prostate tumor cells upregulates expression of neuroendocrine genes, but does not reduce the inhibitory effects of enzalutamide. J. Cell Physiol. 235, 3731–3740. 10.1002/jcp.29267 31587305 PMC6961844

[B79] Mollaoglu G. Guthrie M. R. Bohm S. Bragelmann J. Can I. Ballieu P. M. (2017). MYC drives progression of small cell lung cancer to a variant neuroendocrine subtype with vulnerability to Aurora kinase inhibition. Cancer Cell 31, 270–285. 10.1016/j.ccell.2016.12.005 28089889 PMC5310991

[B80] Mu P. Zhang Z. Benelli M. Karthaus W. R. Hoover E. Chen C. C. (2017). SOX2 promotes lineage plasticity and antiandrogen resistance in TP53- and RB1-deficient prostate cancer. Science 355, 84–88. 10.1126/science.aah4307 28059768 PMC5247742

[B81] Narayanan A. Gagliardi F. Gallotti A. L. Mazzoleni S. Cominelli M. Fagnocchi L. (2019). The proneural gene ASCL1 governs the transcriptional subgroup affiliation in glioblastoma stem cells by directly repressing the mesenchymal gene NDRG1. Cell Death Differ. 26, 1813–1831. 10.1038/s41418-018-0248-7 30538287 PMC6748080

[B82] Nouruzi S. Ganguli D. Tabrizian N. Kobelev M. Sivak O. Namekawa T. (2022). ASCL1 activates neuronal stem cell-like lineage programming through remodeling of the chromatin landscape in prostate cancer. Nat. Commun. 13, 2282. 10.1038/s41467-022-29963-5 35477723 PMC9046280

[B83] Ooi L. Wood I. C. (2007). Chromatin crosstalk in development and disease: Lessons from REST. Nat. Rev. Genet. 8, 544–554. 10.1038/nrg2100 17572692

[B84] Park J. W. Lee J. K. Sheu K. M. Wang L. Balanis N. G. Nguyen K. (2018). Reprogramming normal human epithelial tissues to a common, lethal neuroendocrine cancer lineage. Science 362, 91–95. 10.1126/science.aat5749 30287662 PMC6414229

[B85] Puca L. Gavyert K. Sailer V. Conteduca V. Dardenne E. Sigouros M. (2019). Delta-like protein 3 expression and therapeutic targeting in neuroendocrine prostate cancer. Sci. Transl. Med. 11, eaav0891. 10.1126/scitranslmed.aav0891 30894499 PMC6525633

[B86] Raina K. Lu J. Qian Y. Altieri M. Gordon D. Rossi A. M. (2016). PROTAC-induced BET protein degradation as a therapy for castration-resistant prostate cancer. Proc. Natl. Acad. Sci. U. S. A. 113, 7124–7129. 10.1073/pnas.1521738113 27274052 PMC4932933

[B87] Rotinen M. You S. Yang J. Coetzee S. G. Reis-Sobreiro M. Huang W. C. (2018). ONECUT2 is a targetable master regulator of lethal prostate cancer that suppresses the androgen axis. Nat. Med. 24, 1887–1898. 10.1038/s41591-018-0241-1 30478421 PMC6614557

[B88] Ruan L. Wang L. Wang X. He M. Yao X. (2018). SIRT1 contributes to neuroendocrine differentiation of prostate cancer. Oncotarget 9, 2002–2016. 10.18632/oncotarget.23111 29416748 PMC5788616

[B89] Rudin C. M. Poirier J. T. Byers L. A. Dive C. Dowlati A. George J. (2019). Molecular subtypes of small cell lung cancer: A synthesis of human and mouse model data. Nat. Rev. Cancer 19, 289–297. 10.1038/s41568-019-0133-9 30926931 PMC6538259

[B90] Ryu S. Mcdonnell K. Choi H. Gao D. Hahn M. Joshi N. (2013). Suppression of miRNA-708 by polycomb group promotes metastases by calcium-induced cell migration. Cancer Cell 23, 63–76. 10.1016/j.ccr.2012.11.019 23328481

[B91] Saini S. Majid S. Shahryari V. Arora S. Yamamura S. Chang I. (2012). miRNA-708 control of CD44(+) prostate cancer-initiating cells. Cancer Res. 72, 3618–3630. 10.1158/0008-5472.CAN-12-0540 22552290

[B92] Scher H. I. Fizazi K. Saad F. Taplin M. E. Sternberg C. N. Miller K. (2012). Increased survival with enzalutamide in prostate cancer after chemotherapy. N. Engl. J. Med. 367, 1187–1197. 10.1056/NEJMoa1207506 22894553

[B93] Shafran J. S. Andrieu G. P. Gyorffy B. Denis G. V. (2019). BRD4 regulates metastatic potential of castration-resistant prostate cancer through AHNAK. Mol. Cancer Res. 17, 1627–1638. 10.1158/1541-7786.MCR-18-1279 31110158 PMC6677600

[B94] Shafran J. S. Jafari N. Casey A. N. Gyorffy B. Denis G. V. (2021). BRD4 regulates key transcription factors that drive epithelial-mesenchymal transition in castration-resistant prostate cancer. Prostate Cancer Prostatic Dis. 24, 268–277. 10.1038/s41391-020-0246-y 32690869 PMC7855805

[B95] Shan J. Al-Muftah M. A. Al-Kowari M. K. Abuaqel S. W. J. Al-Rumaihi K. Al-Bozom I. (2019). Targeting Wnt/EZH2/microRNA-708 signaling pathway inhibits neuroendocrine differentiation in prostate cancer. Cell Death Discov. 5, 139. 10.1038/s41420-019-0218-y 31583122 PMC6768854

[B96] Shapiro E. Huang H. Ruoff R. Lee P. Tanese N. Logan S. K. (2008). The heterochromatin protein 1 family is regulated in prostate development and cancer. J. Urol. 179, 2435–2439. 10.1016/j.juro.2008.01.091 18436254

[B97] Shen M. M. Abate-Shen C. (2010). Molecular genetics of prostate cancer: New prospects for old challenges. Genes Dev. 24, 1967–2000. 10.1101/gad.1965810 20844012 PMC2939361

[B98] Shimojo M. Shudo Y. Ikeda M. Kobashi T. Ito S. (2013). The small cell lung cancer-specific isoform of RE1-silencing transcription factor (REST) is regulated by neural-specific Ser/Arg repeat-related protein of 100 kDa (nSR100). Mol. Cancer Res. 11, 1258–1268. 10.1158/1541-7786.MCR-13-0269 23928058

[B99] Shiota M. Song Y. Yokomizo A. Tada Y. Kuroiwa K. Eto M. (2010). Human heterochromatin protein 1 isoform HP1beta enhances androgen receptor activity and is implicated in prostate cancer growth. Endocr. Relat. Cancer 17, 455–467. 10.1677/ERC-09-0321 20308360

[B100] Siegel R. L. Miller K. D. Fuchs H. E. Jemal A. (2022). Cancer statistics, 2022. CA Cancer J. Clin. 72, 7–33. 10.3322/caac.21708 35020204

[B101] Smith B. A. Balanis N. G. Nanjundiah A. Sheu K. M. Tsai B. L. Zhang Q. (2018). A human adult stem cell signature marks aggressive variants across epithelial cancers. Cell Rep. 24, 3353–3366. 10.1016/j.celrep.2018.08.062 30232014 PMC6382070

[B102] Smith B. A. Sokolov A. Uzunangelov V. Baertsch R. Newton Y. Graim K. (2015). A basal stem cell signature identifies aggressive prostate cancer phenotypes. Proc. Natl. Acad. Sci. U. S. A. 112, E6544–E6552. 10.1073/pnas.1518007112 26460041 PMC4664352

[B103] Tan H. L. Sood A. Rahimi H. A. Wang W. Gupta N. Hicks J. (2014). Rb loss is characteristic of prostatic small cell neuroendocrine carcinoma. Clin. Cancer Res. 20, 890–903. 10.1158/1078-0432.CCR-13-1982 24323898 PMC3931005

[B104] Thoma C. (2019). Targeting DLL3 in neuroendocrine prostate cancer. Nat. Rev. Urol. 16, 330. 10.1038/s41585-019-0190-6 31040438

[B105] Vasconcelos F. F. Castro D. S. (2014). Transcriptional control of vertebrate neurogenesis by the proneural factor Ascl1. Front. Cell Neurosci. 8, 412. 10.3389/fncel.2014.00412 25520623 PMC4251449

[B106] Vias M. Massie C. E. East P. Scott H. Warren A. Zhou Z. (2008). Pro-neural transcription factors as cancer markers. BMC Med. Genomics 1, 17. 10.1186/1755-8794-1-17 18489756 PMC2413260

[B107] Wang Z. Wang T. Hong D. Dong B. Wang Y. Huang H. (2022). Single-cell transcriptional regulation and genetic evolution of neuroendocrine prostate cancer. iScience 25, 104576. 10.1016/j.isci.2022.104576 35789834 PMC9250006

[B108] Watson P. A. Arora V. K. Sawyers C. L. (2015). Emerging mechanisms of resistance to androgen receptor inhibitors in prostate cancer. Nat. Rev. Cancer 15, 701–711. 10.1038/nrc4016 26563462 PMC4771416

[B109] Yasumizu Y. Rajabi H. Jin C. Hata T. Pitroda S. Long M. D. (2020). Author correction: MUC1-C regulates lineage plasticity driving progression to neuroendocrine prostate cancer. Nat. Commun. 11, 1095. 10.1038/s41467-020-14808-w 32094369 PMC7039914

[B110] Zhang S. Cui W. (2014). Sox2, a key factor in the regulation of pluripotency and neural differentiation. World J. Stem Cells 6, 305–311. 10.4252/wjsc.v6.i3.305 25126380 PMC4131272

[B111] Zhang Y. Zheng D. Zhou T. Song H. Hulsurkar M. Su N. (2018). Androgen deprivation promotes neuroendocrine differentiation and angiogenesis through CREB-EZH2-TSP1 pathway in prostate cancers. Nat. Commun. 9, 4080. 10.1038/s41467-018-06177-2 30287808 PMC6172226

[B112] Zhou Z. Flesken-Nikitin A. Corney D. C. Wang W. Goodrich D. W. Roy-Burman P. (2006). Synergy of p53 and Rb deficiency in a conditional mouse model for metastatic prostate cancer. Cancer Res. 66, 7889–7898. 10.1158/0008-5472.CAN-06-0486 16912162

